# Determinants of Bone and Blood Lead Levels among Minorities Living in the Boston Area

**DOI:** 10.1289/ehp.6705

**Published:** 2004-05-03

**Authors:** Charles Lin, Rokho Kim, Shirng-Wern Tsaih, David Sparrow, Howard Hu

**Affiliations:** ^1^School of Medicine, University of California at San Francisco, San Francisco, California, USA; ^2^Occupational Health Surveillance Program, Massachusetts Department of Public Health, Boston, Massachusetts, USA; ^3^Channing Laboratory, Department of Medicine, Brigham and Women’s Hospital, Harvard Medical School, Boston, Massachusetts, USA; ^4^Occupational Health Program, Department of Environmental Health, Harvard School of Public Health, Boston, Massachusetts, USA; ^5^Statistical Genetics Group, Jackson Laboratories, Bar Harbor, Maine, USA; ^6^Normative Aging Study, Department of Veterans Affairs Medical Center, Boston, Massachusetts, USA

**Keywords:** blacks, blood lead, bone lead, minority groups, occupations, smoking, X-ray fluorescence

## Abstract

We measured blood and bone lead levels among minority individuals who live in some of Boston’s neighborhoods with high minority representation. Compared with samples of predominantly white subjects we had studied before, the 84 volunteers in this study (33:67 male:female ratio; 31–72 years of age) had similar educational, occupational, and smoking profiles and mean blood, tibia, and patella lead levels (3 μg/dL, 11.9 μg/g, and 14.2 μg/g, respectively) that were also similar. The slopes of the univariate regressions of blood, tibia, and patella lead versus age were 0.10 μg/dL/year (*p* < 0.001), 0.45 μg/g/year (*p* < 0.001), and 0.73 μg/g/year (*p* < 0.001), respectively. Analyses of smoothing curves and regression lines for tibia and patella lead suggested an inflection point at 55 years of age, with slopes for subjects ≥ 55 years of age that were not only steeper than those of younger subjects but also substantially steeper than those observed for individuals > 55 years of age in studies of predominantly white participants. This apparent racial disparity at older ages may be related to differences in historic occupational and/or environmental exposures, or possibly the lower rates of bone turnover that are known to occur in postmenopausal black women. The higher levels of lead accumulation seen in this age group are of concern because such levels have been shown in other studies to predict elevated risks of chronic disease such as hypertension and cognitive dysfunction. Additional research on bone lead levels in minorities and their socioeconomic and racial determinants is needed.

Research has suggested that lead toxicity may disproportionately affect minority groups. (Bailey et al. 1994; Rothenberg et al. 1999). Despite substantial declines in blood lead levels in the general U.S. population, a substantial body of research, including data from the Third National Health and Nutrition Examination Survey (NHANES III), shows that African Americans continue to have higher blood lead levels than do whites (Brody et al. 1994; Lanphear et al. 1996; Mahaffey et al. 1982; Pirkle et al. 1998).

Most studies analyzing racial differences in lead toxicity have focused on blood lead as a biomarker. Although blood lead mostly provides an accurate measure of recent lead exposure, evidence has been growing to indicate that this biomarker does not adequately reflect an individual’s health risk due to cumulative lead exposure (Hu et al. 1998). In adults, about 95% of the total body lead burden is stored in the skeleton (Barry and Mossman 1970), and the half-life of lead in bone ranges from years to decades (Rabinowitz 1991). With a half-life of up to 25 years (Rabinowitz et al. 1976), bone lead is a biologic marker of cumulative lead exposure over many years and may better predict the effects of lead toxicity that arise from chronic low to moderate exposure, such as hypertension (Cheng et al. 2001; Glenn et al. 2003; Hu et al. 1996a; Korrick et al. 1999; Lee et al. 2001).

Sociodemographic rather than genetic factors, including low income and residence in older housing, have been attributed to the higher blood lead levels seen in black children (Pirkle et al. 1998). Although low income and education have also been correlated with higher blood and bone lead levels among white males (Elreedy et al. 1999; Hu et al. 1996b), minority groups are disproportionately affected. In recent data from the Normative Aging Study, nonwhite blue-collar workers had significantly higher blood and patella lead did than white blue-collar workers, suggesting an interaction between occupational exposures and race/ethnicity (Elmarsafawy et al. 2002). However, the generalizability of this study is limited by its size, because nonwhites comprised < 2% of the total sample population. To build on this work, in the Community Lead Study we focused on analyzing the bone lead levels of an exclusively minority sample.

## Materials and Methods

### Study subjects.

Subjects were recruited from the pool of subjects who participated in a study funded by the National Institutes of Health (NIH; “Impact of Sleep-Disordered Breathing in Older Adults,” NIH HL51075; principal investigator, D. Sparrow; 1 July 1994–30 June 1997) that had significant minority and female involvement. These subjects had been initially recruited via solicitation letters sent to residents in Boston, Massachusetts, census tracts in the Jamaica Plain neighborhood with high minority representation. Additional subjects for our study were drawn from the Roxbury, Dorchester, and Jamaica Plain neighborhoods—which also have high minority representation—through participant referrals to family members and friends.

Letters introducing the study and demographic and consent forms were sent to potential subjects. Only minority subjects ≥ 35 years of age were accepted. Willing and eligible participants were invited to the Brigham and Women’s Hospital outpatient clinic in Boston, where a fresh whole blood specimen was collected for lead measurement and where K X-ray fluorescence (KXRF) bone lead measurements were taken. Blood and bone lead measurements were taken between 1999 and 2000 for participating subjects. Participants who completed the study were reimbursed for their time and effort.

The human research committees of the Brigham and Women’s Hospital and the Department of Veterans Affairs Medical Center in Boston approved the research project. Written informed consent was obtained from all participants.

### Blood lead measurement.

Blood for lead measurements was collected in 7-mL trace-metal–free tubes (Becton-Dickinson Co., Bedford, MA) containing EDTA and sent for analysis to ESA Laboratories, Inc. (Chelmsford, MA). The ESA Laboratories blood lead analysis protocol and quality control and quality assurance specifications are described elsewhere (Hu et al. 1996b).

### KXRF bone lead measurement.

An ABIOMED KXRF instrument (ABIOMED, Inc., Danvers, MA) was used to take bone lead measurements of each subject’s midtibial shaft and patella. The physical principles, technical specifications, validation, and quality control procedures of this (Burger et al. 1990; Hu et al. 1990, 1994) and similar KXRF instruments (Ellis et al. 1987; Somervaille et al. 1985) have been described in detail elsewhere.

Briefly, KXRF uses a ^109^Cd gamma-ray source to induce fluorescence from the target tissue. The emitted photons are then detected, counted, and arrayed on a spectrum (Hu et al. 1989). The net lead signal is determined after Compton background counts are subtracted by a linear least-squares algorithm.

For each subject, 30-min measurements were taken at the midshaft of the tibia and patella after each region had been washed with a 70% solution of isopropyl alcohol. The KXRF beam collimator was sited perpendicular to the flat bone surface for the tibia and patella.

### Statistical analyses.

We used Stata version 7.0 (Stata Corporation, College Station, TX) and S-Plus version 6.1 (Insightful Corporation, Seattle, WA) for database management and statistical analysis. The quality of the KXRF measurements was preserved by discarding tibia and patella lead values with associated measurement-uncertainty estimates of > 10 μg/g and > 15 μg/g, respectively. Negative tibia and patella measurements were retained to minimize bias and increase efficiency of comparing bone lead levels among different populations (Kim et al. 1995).

We created final education categories after collapsing comparable educational levels that had similar blood and bone lead data. Subjects with technical school training and college education were pooled together, as were students with graduate and professional schooling. For race, the 69 black participants comprised one category and the 15 other subjects, who were Hispanic, Asian, and American Indian, were classified as “other.” For job type, we classified retired subjects as white collar or blue collar based on their previous occupation. For example, doctors, lawyers, engineers, and so on, were categorized as white collar, whereas technicians, repairmen, carpenters, and so forth, were categorized as blue collar. We adopted a complete classification list of professions which has been published elsewhere by Elmarsafawy et al. (2002).

We examined blood, patella, and tibia lead levels across categories of age, race, education, smoking status, alcohol consumption, and job type. Simple linear regression of blood, tibia, and patella lead level versus age was performed over the entire age range. We performed graphic evaluation by locally weighted scatter plot smoothing (Lowess) to verify and select an inflection point for both biomarkers (Cleveland et al. 1976). Separate regression analyses were performed on subjects younger and older than this cutoff point, and male and female data were analyzed separately and together.

Multiple linear regression models were constructed to predict blood, tibia, and patella lead. Age, sex, race, educational level, alcohol consumption, cumulative smoking, and job type—variables known to be associated with these biomarkers—were forced into all models. Interaction terms of black race with blue-collar work, black race with male sex, and male sex with blue-collar work were tested for significance.

## Results

A total of 108 subjects participated in this study, coming from the Jamaica Plain, Roxbury, and Dorchester neighborhoods in Boston: 86 black, 7 Hispanic, 3 American Indian, 2 Asian, and 10 other or unknown. Response rates to mailings in the parent study (< 10%) and the present study (< 10%) made our sample largely one of convenience. A final population of 84 subjects were included in the present analyses after we excluded 14 subjects for tibia (*n* = 13) and patella (*n* = 1) lead values associated with measurement uncertainty estimates of > 10 μg/g and > 15 μg/g, respectively, and 10 subjects who were missing covariate values for education (*n* = 6) and/or job type (*n* = 4). Comparisons between the 84 included subjects and 24 excluded subjects revealed no meaningful differences with regard to blood, tibia, or patella lead or age, race, education, pack-years of smoking, alcohol consumption, or job type.

Our sample of 84 subjects had a mean age of 50 years (range, 31–77 years), and 56 (67%) subjects were female (Table 1). The proportions of subjects whose education was limited to high school or lower (48%), who had a history of smoking (57%), and who worked in blue-collar jobs (33%) were not too dissimilar from the proportions we observed among the predominantly white subjects participating in the Normative Aging Study (47, 68, and 41%, respectively; Hu et al. 1996b; Elmarsafawy et al. 2002).

**Figure 1:**
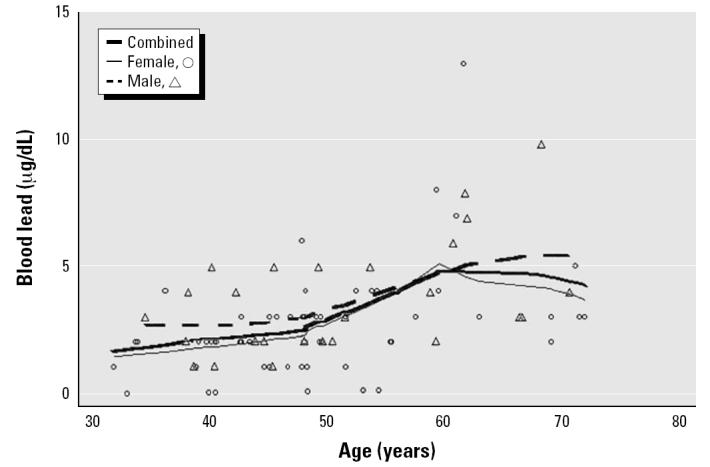
Scatter plots and smoothed lines of blood lead levels (*n* = 84) versus age in community-exposed minority subjects, Community Lead Study, Boston, Massachusetts, 1999–2000.

**Figure 2:**
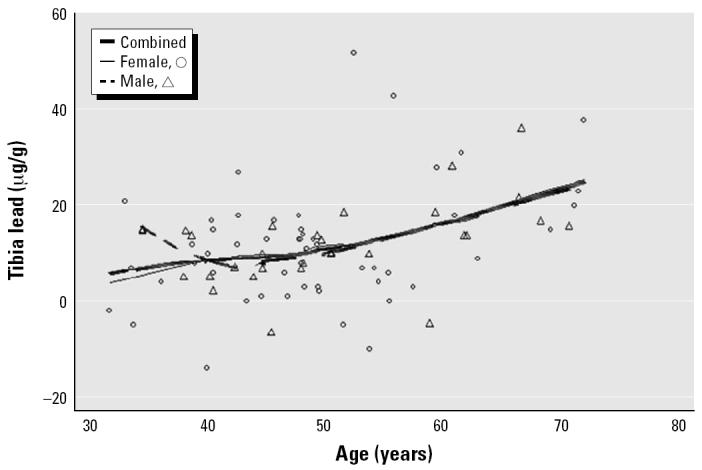
Scatter plots and smoothed lines of tibia lead levels (*n* = 84) versus age in community-exposed minority subjects, Community Lead Study, Boston, Massachusetts, 1999–2000.

**Figure 3:**
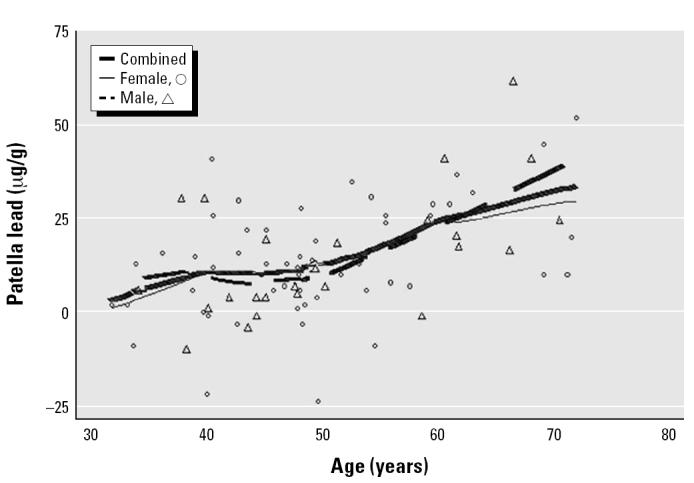
Scatter plots and smoothed lines of patella lead levels (*n* = 84) versus age in community-exposed minority subjects, Community Lead Study, Boston, Massachusetts, 1999–2000.

**Figure 4:**
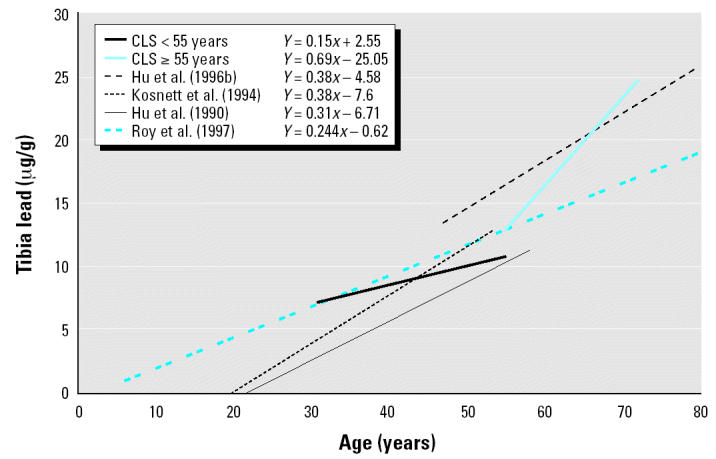
Comparison of regression lines of tibia bone lead versus age between community-exposed minority subjects from the Community Lead Study, Boston, Massachusetts, 1999–2000, and other studies of predominantly white participants.

The mean and median blood lead levels of the present sample of 84 subjects were 3.0 μg/dL and 2.2 μg/dL, respectively. The mean ± SD for tibia lead was 11.9 ± 11.0 μg/g, and for patella lead, 14.2 ± 15.3 μg/g. In simple regression models, the slope coefficients of blood lead, and patella lead versus age were 0.10 μg/dL/year (*p* < 0.001), 0.45 μg/g/year (*p* < 0.001), and 0.73 μg/g/year (*p* < 0.001), respectively. Further analysis with smoothing plots indicated that the associations between age and bone lead biomarkers were nonlinear. In general, the univariate regression slopes of tibia and patella lead versus age were greater among subjects ≥ 55 years of age than among those < 55 years of age (Figures 1–3). A simple linear regression of patella lead versus age followed an average slope of 0.2 μg/g/year up to 55 years and then inflected upward to increase at 0.83 μg/g/year in subjects ≥ 55 years of age (data not shown). Likewise, tibia lead increased at a rate of 0.15 μg/g/year in participants < 55 years of age and at a rate of 0.69 μg/g/year ≥ 55 years of age (Figure 4). The differences in regression coefficients between the two age groups were statistically significant. Because of limited published data on patella lead, only the regression coefficients of tibia lead are compared with those observed in other studies in Figure 4 (Hu et al. 1990; Hu et al. 1996b; Kosnett et al. 1994; Roy et al. 1997).

For blood and patella lead, males and females < 55 years of age had similar rates of lead accumulation with increasing age; in contrast, among subjects ≥ 55 years of age, males had higher rates of accumulation. For tibia lead, a similar trend was observed, but the disparity among those ≥ 55 years was smaller.

In multiple regression models with independent variables that included age, sex, race, pack-years of smoking, drinking, educational levels, and occupation, age was the most significant predictor for blood, tibia, and patella lead (Table 2). A history of smoking > 20 pack-years predicted a 7.2 μg/g/year increase in tibia lead with borderline significance (*p* < 0.10). Having a blue-collar occupation significantly predicted an 8.02 μg/g increase in patella lead (*p* < 0.05). When interaction terms between black race and blue-collar work, black race and male sex, and male sex and blue-collar occupation were tested as predictors of lead biomarkers, they were insignificant (data not shown).

## Discussion

The blood and bone lead levels we observed in this study indicate that this minority sample had lead exposure similar to that of the general population. The relatively low levels of blood lead (mean, 3.0 μg/dL) parallel those reported for individuals 20–74 years of age in the 1988–1991 NHANES III (mean, 3.0 μg/dL) (Pirkle et al. 1994). Studies of community-exposed, predominantly white subjects of similar age had tibia lead levels (Gamblin et al. 1994; Kosnett et al. 1994; Somervaille et al. 1988) and patella lead levels (Korrick et al. 2002) comparable with those observed in this study.

As seen in other studies of general population samples, age was the predominate correlate of tibia lead (Gamblin et al. 1994; Kosnett et al. 1994; McNeill et al. 2000) and patella lead (Hu et al. 1996b; Korrick et al. 2002), accounting for nearly half of the variability in multivariate regressions of both biomarkers. The strong association between age and bone lead probably reflects exposure to different levels of environmental lead over time, that is, the birth cohort effect previously reported (Kim et al. 1997). Univariate smoothing curves and simple regression models of tibia and patella lead versus age showed a smaller slope among subjects < 55 years of age, an inflection point at 55 years, and a greater slope at ≥ 55 years (Figures 2 and 3). These results may reflect a trend similar to that observed previously for tibia lead among community-exposed white men (Kosnett et al. 1994). We did not attempt a nonlinear model, which would likely have overfitted data from our small sample size.

Compared with age-related increases in bone lead of β = 0.31 μg/g/year observed by Hu et al. (1990) and β = 0.38 μg/g/year observed by Kosnett et al. (1994), tibia lead in our study increased at a lower rate (β = 0.15 μg/g/year) for subjects < 55 years of age. This discrepancy may be due to the differences in age range among these three studies. Study subjects from both the Hu et al. (1990) and Kosnett et al. (1994) studies, with ranges of 21–58 years and 20–55 years, respectively, spanned a wider age range, including younger individuals in their third decade with higher growth and bone formation rates.

In contrast, among subjects ≥ 55 years of age, tibia lead increased at a greater rate (β = 0.69 μg/g/year) than that measured in previous Normative Aging Study research (β = 0.38 μg/g/year; Hu et al. 1996b) (Figure 4). It is possible that genetic differences in bone turnover account for some of the observed discrepancy in those subjects ≥ 55 years of age. Two studies (Aloia et al. 1998; Luckey et al. 1996) have shown that postmenopausal black women have lower rates of bone turnover than do postmenopausal white women. Decreased formation of new bone in a more recent lower-lead environment would result in higher relative bone lead levels. The fact that women comprise two-thirds of our subjects may explain why the tibia lead accumulation rate in subjects ≥ 55 years of age was higher than that observed elsewhere (Hu et al. 1996b).

The steeper bone lead–age relationship in older individuals may also be due to increased environmental exposures among retirees. Boston’s prevalence of old housing has been tied to greater lead exposure via dust inhalation from lead paint in children (Bailey et al. 1994) and via ingestion of water contaminated from lead plumbing in adults (Potula et al. 1999). Housing age and lead amounts in tap water were not measured in this study, but given the fact that approximately 50% of housing in Boston was built before 1950 (Massachusetts Department of Public Health 1999), it seems likely that retirees who spend more time at home are at increased risk for environmental lead exposure.

When analyzed by sex, males exhibited a higher rate of blood and patella lead accumulation among subjects ≥ 55 years of age. This sex-related difference may be due to increased bone remodeling in postmenopausal women, supporting a trend previously reported by Hertz-Picciotto et al. (2000) and Rothenberg et al. (1994). As old bone is replaced by new bone matrix formed in a more recent lower-lead environment, women may experience a relative decrease in bone lead concentration. The same trend is blunted in tibia lead and reflects its lower bone turnover rate and thus decreased sensitivity to the onset of menopause.

Although participants were not asked if they had occupational lead exposure, in multivariate analyses blue-collar work by itself was a significant determinant of patella lead (*p* < 0.05). In previous research of a sample of white men who were not employed in lead-related industries, we found that tibia and patella lead levels were higher in those employed in blue-collar jobs (Elmarsafawy et al. 2002). Because the same classification criteria for white-collar and blue-collar jobs were used in this study, this analysis provides some support that occupational lead exposure is a risk factor for these minority individuals. Unlike our findings in the Normative Aging Study (Hu et al. 1996b), low education was not a significant predictor of blood or bone lead; however, individuals in our sample were relatively well educated, with > 50% having had some college education and two-thirds working in white-collar jobs, which may have limited our ability to discern the influence education as a proxy of social class.

The main limitations of this study stem from our relatively limited sample size as well as potential biases related to our subject recruitment. Although our subjects came from some of Boston’s high-minority-representation communities, they were volunteers who had participated in previous research and who essentially comprised a convenience sample. In addition, our models of bone lead were only able to explain up to 24% of their variance—a figure that is similar to those found in other studies such as the Normative Aging Study (Hu et al. 1996b); thus, there are likely many unmeasured factors that could explain differences in bone lead distributions. Nevertheless, comparisons of our data with those from the predominantly white subjects participating in the Normative Aging Study and other populations is instructive, particularly because their distributions of the main factors determined to be predictors of bone lead in the general population—education, smoking, and occupational status—are similar. In so doing, our finding of tibia bone lead levels in subjects > 55 years of age that were higher than those seen in whites of similar age is concerning because elevated bone lead levels have been clearly implicated as a risk factor for chronic disease such as hypertension (Cheng et al. 2001; Glenn et al. 2003; Hu et al. 1996a; Korrick et al. 1999; Lee et al. 2001). Clearly, more research is needed to examine bone and blood lead levels in minority groups, with an increased emphasis on those who may have lower educational and occupational status and who may therefore be at greatest risk for chronic lead exposure and its impact on health.

## Figures and Tables

**Table 1:** Lead biomarker levels (mean ± SD) among Community Lead Study subgroups, Boston, Massachusetts, 1999–2000.

Variable	No.	Blood lead (μg/dL)	Tibia lead (μg/g)	Age-adjusted tibia lead (μg/g)	Patella lead (μg/g)	Age-adjusted patella lead (μg/g)
Age (years)						
< 45	28	2.0 ± 1.2	8.3 ± 8.4		8.9 ± 14.3	
46–60	41	2.8 ± 1.7	10.8 ± 11.5		11.8 ± 11.4	
61–75	15	5.3 ± 3.2*^#^*	21.7 ± 8.6*^#^*		30.9 ± 15.7*^#^*	
Sex						
Female	56	2.7 ± 2.2	11.8 ± 11.9	12.0 ± 11.0	13.8 ± 15.0	14.1 ± 13.5
Male	28	3.6 ± 2.2*	12.1 ± 9.0	11.7 ± 7.9	15.0 ± 16.1	14.3 ± 13.3
Race						
Black	69	3.0 ± 2.3	12.9 ± 11.2	12.7 ± 10.4	14.6 ± 15.4	14.4 ± 12.9
Other	15	2.9 ± 2.0	7.3 ± 8.8*	7.9* ± 7.2	12.3 ± 14.9	13.3 ± 15.9
Education						
High school dropout	14	2.6 ± 2.2	9.4 ± 6.3	11.3 ± 7.4	12.9 ± 10.0	16.0 ± 10.1
High school graduate	26	3.6 ± 2.9	13.7 ± 12.9	12.9 ± 10.8	16.9 ± 20.3	15.7 ± 16.4
Technical school, college	31	2.7 ± 1.4	10.8 ± 9.2	10.8 ± 8.9	12.2 ± 12.0	12.2 ± 11.0
Graduate school, professional	13	3.0 ± 2.1	13.6 ± 14.8	13.0 ± 13.8	15.2 ± 16.0	14.1 ± 15.8
Smoking (pack-years)						
0	36	2.7 ± 1.9	9.9 ± 11.1	9.9 ± 11.0	12.7 ± 14.9	12.7 ± 14.6
1–19	38	3.0 ± 2.1	11.9 ± 10.3	12.4 ± 8.7	12.5 ± 14.4	13.3 ± 11.5
≥ 20	10	4.1 ± 3.3	19.2 ± 11.3*	17.5 ± 9.5*	26.0 ± 16.1**	23.2 ± 13.1*
Consuming ≥ 2 alcoholic drinks/day						
No	78	2.9 ± 2.2	12.0 ± 11.2	11.9 ± 10.2	14.0 ± 15.6	13.8 ± 13.6
Yes	6	3.7 ± 2.5	10.0 ± 7.9	12.0 ± 8.9	16.3 ± 11.3	19.5 ± 9.8
Job type						
White collar/mixed	56	2.8 ± 2.1	11.3 ± 11.7	11.1 ± 10.9	12.0 ± 15.3	11.8 ± 13.3
Blue collar	28	3.4 ± 2.3	13.1 ± 9.6	13.4 ± 8.1	18.6 ± 14.5*	19.1 ± 12.5**

* *p* < 0.1,

** *p* < 0.05, and

# *p* < 0.01 by analysis of variance.

**Table 2:** Multiple regression of lead biomarker levels in the Community Lead Study, Boston, Massachusetts, 1999–2000.

	Blood lead (μg/dL)		Tibia lead (μg/g)		Patella lead (μg/g)	
Characteristic	β	95% CI	β	95% CI	β	95% CI
Age (years)	0.10	0.05–0.14	0.42	0.19–0.65	0.72	0.42–1.02
Male sex	0.59	−0.46–1.65	−1.75	−7.13–3.63	−4.04	−11.08–3.00
Black race*^a^*	−0.11	−1.28–1.07	4.04	−1.92–10.00	−0.29	−8.09–7.51
Education*^b^*						
High school graduate	0.56	−0.92–2.03	−0.52	−8.03–7.00	−0.17	−9.99–9.66
Technical school, college	0.07	−1.35–1.50	−0.82	−8.08–6.44	−1.40	−10.90–8.10
Graduate school, professional	0.16	−1.47–1.78	2.04	−6.23–10.32	0.38	−10.45–11.21
Smoking (pack-years)*^c^*						
0–20	0.10	−0.89–1.09	2.67	−2.37–7.72	−0.53	−7.13–6.07
≥ 20	0.52	−1.07–2.11	7.18	−0.92–15.29	8.62	−1.98–19.22
Currently consuming	0.97	−0.85–2.80	−1.41	−10.72–7.90	1.12	−11.06–13.31
≥ 2 alcoholic drinks/day						
Blue-collar occupation*^d^*	0.22	−0.92–1.35	2.10	−3.67–7.87	8.02	0.47–15.58
Total model adjusted *R*^2^	0.18		0.15		0.24	

CI, confidence interval.

a Compared with other minorities.

b Baseline: high school dropout.

c Compared with 0 pack-years of smoking.

d Compared with white-collar occupations.
